# Pediatric Thyroidectomy: Experience From a Portuguese Hospital

**DOI:** 10.7759/cureus.33259

**Published:** 2023-01-02

**Authors:** Paula Manuel Vieira, Joana Barbosa Sequeira, Sílvia Santos Monteiro, Ana De Carvalho Vaz, Juliana da Silva Cardoso, Luís Ribeiro, Catarina Mendes, Joana Freitas, João Ribeiro de Castro, Teresa Borges, Maria João Oliveira

**Affiliations:** 1 Pediatrics, Centro Materno-Infantil do Norte Albino Aroso, Centro Hospitalar Universitário do Porto, Porto, PRT; 2 Pediatric Surgery, Centro Materno-Infantil do Norte Albino Aroso, Centro Hospitalar Universitário do Porto, Porto, PRT; 3 Division of Endocrinology, Diabetes and Metabolism, Centro Hospitalar Universitário do Porto, Porto, PRT; 4 Pediatrics, Unidade Local de Saúde do Alto Minho, Viana do Castelo, PRT; 5 Pediatrics, Unidade Local de Saúde do Nordeste, Bragança, PRT; 6 Pediatric Endocrinology, Centro Materno-Infantil do Norte Albino Aroso, Centro Hospitalar Universitário do Porto, Porto, PRT

**Keywords:** thyroid pathology, pediatrics, thyroid diseases, thyroidectomy, thyroid nodule

## Abstract

Background and objective

Pediatric thyroid disease requiring surgery is rare. Thyroid nodules are a frequent indication for surgery and are mostly benign. However, up to 25% of cases can be malignant. In this study, we aimed to describe our center’s experience with regard to pediatric thyroid surgery.

Methods

This was a retrospective transverse study involving pediatric patients who underwent thyroid surgery at a tertiary hospital between January 2010 and December 2021.

Results

A total of 14 patients underwent 15 surgeries. The main reason for referral to pediatric endocrinology was thyroid nodules (n=10). Thirteen fine needle aspirations (FNAs) were performed, with follicular tumor (n=6) being the most common finding. The median age of patients at surgery was 15.9 years [interquartile range (IQR): 14.0-16.8]. The most common surgical indications were the presence of a follicular tumor on FNA (n=5) and thyroid nodule size causing symptoms (n=5). There was one case of prophylactic thyroidectomy due to the identification of a multiple endocrine neoplasia type 2A (MEN2A) mutation. The most frequently described histopathology results were follicular adenoma (n=6) and colloid nodular goiter (n=6). Three postoperative complications were observed in three different patients: bilateral lesion of the recurrent laryngeal nerve, cervical hematoma, and transient hypoparathyroidism with hypocalcemia.

Conclusion

In our study, the most frequent surgical indication was a follicular tumor. A good correlation was found between FNA cytology and final histopathology results, which is in accordance with previous studies. This reinforces the importance of FNA in diagnosis and surgical planning. The rate of complications in our study is comparable to that in larger single-center series in the literature.

## Introduction

Pediatric thyroid disease requiring surgery is rare, and hence there is scarce data on surgical experience in pediatric thyroid disease [1-3]. Hypothyroidism, Graves disease, and thyroid nodules are the most common thyroid disorders in children [4,5]. Based on the available literature, the most common indications for thyroid surgery are thyroid nodules for histological diagnosis and the removal of macroscopic disease [1-3].

Palpable thyroid nodules are present in ~2% of children [4,6]. Their frequency is equally distributed between both genders before the age of 10 years but becomes twice as common in females after this age [6]. The majority of these are benign but up to 25% can be malignant, which is a larger proportion compared to adult patients [4,6,7]. Thus, it is important to differentiate between malignant and benign diseases and identify those who require surgery [8]. Some ultrasonographic features are associated with an increased risk of thyroid cancer, such as irregular shape and margins, microcalcifications, marked hypoechogenicity, intranodular hypervascularization, enlargement of the nodule over time, invasion of extra thyroid tissues, anteroposterior diameter larger than the transverse diameter, and the presence of cervical adenopathies [4,6,9,10]. Clinical criteria that are associated with a higher risk of malignant disease are a family history of thyroid cancer or multiple endocrine neoplasia (MEN) in first-degree relatives, age below 14 years, exposure to radiation, personal history of diseases associated with a higher risk of thyroid cancer (e.g., Cowden syndrome) or previous surgery for thyroid cancer, the presence of a focal hyperfixation on PET scan, hard and adherent lymph node on palpation, rapid and progressive growth, presence of symptoms associated with compression of adjacent anatomical structures, and the presence of a suspect adenopathy [10]. Fine needle aspiration (FNA) cytology has high diagnostic accuracy and is frequently used to aid in surgical planning [3,9]. However, there are some limitations to the use of this technique in children, such as higher rates of nondiagnostic samples compared to adults and the need for sedation or general anesthesia [8].

The most common surgical complications described are hypoparathyroidism, hypocalcemia, and recurrent laryngeal nerve injury [1,6,10]. Younger age, total thyroidectomy, Graves disease, and malignancy seem to be related to a higher risk of hypocalcemia after thyroid surgery [1-3]. In this study, we aimed to describe our center’s experience in pediatric thyroidectomy.

## Materials and methods

Study design and setting

We conducted a retrospective transverse study involving all pediatric patients who underwent thyroid surgery by pediatric surgeons at a tertiary hospital between January 2010 and December 2021.

Data collection

Demographical and clinical data were collected from available electronic clinical records, including age, gender, presenting symptoms, ultrasound results (presence, size, and characteristics of nodules), thyroid function test, previous medical history, surgical indication and procedure (total or hemithyroidectomy), postoperative complications (hypoparathyroidism, hypocalcemia, and recurrent laryngeal nerve injury), FNA cytology, and final surgical histopathology results. All surgeries were performed by the same experienced surgeon. Data analysis was conducted using Microsoft Office Excel 2016 software. This study was approved by the hospital’s ethics committee with the approval number 2022.114(088-DEFI/090-CE).

## Results

During the study period, a total of 14 patients underwent 15 surgeries. A majority (n=12) were female with a median age of 13.9 years at the first pediatric endocrinology appointment [interquartile range (IQR): 11.0-15.4].

The main reason for referral to pediatric endocrinology was the presence of thyroid nodules in 10 patients. The median size of thyroid nodules was 35.5 mm (IQR: 30.0-46.0). Other reasons for referral included Graves disease and, in one case, the identification of a mutation for multiple endocrine neoplasia type 2A (MEN2A). A majority (n=11) of patients had a normal thyroid function, with one case of hypothyroidism and one of hyperthyroidism. One child presented with subclinical hypothyroidism. Six patients were found to have additional diseases: autoimmune thyroiditis (n=3), chronic kidney disease (n=2), and autism spectrum disorder (n=1).

Thirteen FNA were performed. FNA was not required in two cases: one case of Graves’ disease and the patient with MEN2A mutation. A follicular tumor (n=5) was the most common finding. Table 1 shows FNA results according to the Bethesda 2017 classification. One patient was submitted to scintigraphy, which showed a toxic nodule.

**Table 1:** FNA results according to the Bethesda 2017 classification FNA: fine-needle aspiration

Bethesda classification		N
Bethesda I	Cystic lesion	1
Bethesda II	Colloid nodular goiter	2
	Adenoma	1
	Benign follicular nodule	2
Bethesda III	Atypia of undetermined significance	2
Bethesda IV	Follicular tumor	5

A total of 15 surgeries were performed: six total thyroidectomies (including one completion thyroidectomy) and nine hemithyroidectomies (seven of the right lobe and two of the left lobe). The median age at the time of surgery was 15.9 years (IQR: 14.0-16.8). The most common surgical indications were the presence of a follicular tumor on FNA (n=5), thyroid nodule size causing symptoms (n=5), and atypia of undetermined significance on FNA (n=2). Other indications were present in one patient each, as follows: toxic adenoma, Graves disease, and prophylactic thyroidectomy due to the identification of a mutation for MEN2A.

Final operative histopathology results included follicular adenoma (n=6), colloid nodular goiter (n=6), noninvasive follicular thyroid neoplasm with papillary-like nuclear features (NIFTP) (n=1), papillary carcinoma (n=1), and C cell hyperplasia compatible with MEN2A (n=1). All three patients with autoimmune thyroiditis also had changes compatible with chronic lymphocytic thyroiditis. The correlation between FNA and pathology results is presented in Table 2.

**Table 2:** Association between FNA and pathology results FNA: fine-needle aspiration; NIFTP: noninvasive follicular thyroid neoplasm with papillary-like nuclear features

FNA results (n)	Pathology results (n)
Cystic lesion (1)	Colloid nodular goiter (1)
Colloid nodular goiter (2)	Colloid nodular goiter (1)
	NIFTP (1)
Adenoma (1)	Colloid nodular goiter (1)
Benign follicular nodule (2)	Colloid nodular goiter (2)
Atypia of undetermined significance (2)	Hurtle follicular adenoma (1)
	Papillary carcinoma (1)
Follicular tumors (5)	Follicular adenoma (4)
	Microfollicular follicular adenoma (1)

Three postoperative complications occurred in three different patients: persistent bilateral lesion of the recurrent laryngeal nerve, cervical hematoma without the need for drainage, and transient hypoparathyroidism with hypocalcemia. Both patients in which malignant disease was identified (NIFTP and papillary carcinoma) were transferred to another hospital specializing in oncology for further treatment. All other patients submitted to hemithyroidectomy either maintain follow-up or were transferred to adult care upon turning 18 years of age.

## Discussion

In our study, the most frequent surgical indication was the presence of a follicular tumor on FNA. Literature shows that thyroid nodules are the most common surgical indication without further specification [1,3,7]. In our study, the majority of patients also presented with thyroid nodules. However, nodules by themselves do not represent a surgical indication. Other studies show a higher prevalence of malignancy when compared to ours [1,3]. This may be attributed to the existence of other hospitals in our area specializing in the treatment of malignant diseases.

There was a good correlation between FNA cytology and final surgical histopathology results, which is in accordance with previous studies [1]. This reinforces the importance of FNA in both diagnosis and surgical planning.

Regarding surgical complications, data from other studies vary widely between 1% and 47% [1,7,11]. Our rate of complications is similar (20%) and comparable to other larger single-center series in the literature. Given that hypocalcemia is more common after total thyroidectomy, the fact that hemithyroidectomies were more common in our series may explain why it was only present in one patient.

There are some limitations to our study, such as the small sample size and the single-center design. Retrospective data collection may also have led to data-related bias.

## Conclusions

This study adds to the current data available on the topic, as it is the first paper describing thyroid surgery experience in a Portuguese pediatric population. Given the lack of data on pediatric thyroid surgery, we would like to emphasize the importance of further research in this field.

## References

[1] Almosallam OI, Aseeri A, Alhumaid A, AlZahrani AS, Alsobhi S, AlShanafey S (2020). Thyroid surgery in 103 children in a single institution from 2000-2014. Ann Saudi Med.

[2] Chen Y, Masiakos PT, Gaz RD (2015). Pediatric thyroidectomy in a high volume thyroid surgery center: risk factors for postoperative hypocalcemia. J Pediatr Surg.

[3] Scholz S, Smith JR, Chaignaud B, Shamberger RC, Huang SA (2011). Thyroid surgery at Children's Hospital Boston: a 35-year single-institution experience. J Pediatr Surg.

[4] Hong HS, Lee JY, Jeong SH (2017). Thyroid disease in children and adolescents. Ultrasonography.

[5] Dimachkieh AL, Kazahaya K, Chelius DC Jr (2019). Assessment and management of thyroid disease in children. Otolaryngol Clin North Am.

[6] Wassner AJ (2023). Wassner AJ: thyroid nodules and cancer in children - UpToDate. https://www.uptodate.com/contents/thyroid-nodules-and-cancer-in-children?search=thyroidnodule&source=search_result&selectedTitle=1~150&usage_type=default&display_rank=1.

[7] Wood JH, Partrick DA, Barham HP, Bensard DD, Travers SH, Bruny JL, McIntyre RC Jr (2011). Pediatric thyroidectomy: a collaborative surgical approach. J Pediatr Surg.

[8] Amirazodi E, Propst EJ, Chung CT, Parra DA, Wasserman JD (2016). Pediatric thyroid FNA biopsy: outcomes and impact on management over 24 years at a tertiary care center. Cancer Cytopathol.

[9] Mussa A, De Andrea M, Motta M, Mormile A, Palestini N, Corrias A (2015). Predictors of malignancy in children with thyroid nodules. J Pediatr.

[10] Direção-Geral da Saúde (2023). Diagnostic approach to thyroid nodule in pediatrics and adults (Portuguese). Abordagem Diagnóstica do Nódulo da Tiroide em Idade Pediátrica e no Adulto. Direção-Geral da Saúde. Published online.

[11] Dream S, Wang R, Lovell K, Iyer P, Chen H, Lindeman B (2020). Outpatient thyroidectomy in the pediatric population. Am J Surg.

